# Wearable devices for postoperative monitoring in surgical ward and the chain of liability

**DOI:** 10.1186/s44158-024-00154-6

**Published:** 2024-03-07

**Authors:** Valentina Bellini, Marco Brambilla, Elena Bignami

**Affiliations:** 1 Department of Medicine and Surgery, Anesthesiology, Critical Care and Pain Medicine Division, University of Parma, Viale Gramsci 14, Parma, 43126 Italy; 2 Department of Information and Communication Technology, University Hospital of Parma, Parma, Italy

**Keywords:** Wearable devices, Perioperative medicine, Postoperative monitoring, Surgical ward, Artificial intelligence

## Abstract

Perioperative medicine is undergoing many changes with the introduction of new technologies. Wearable devices are among them. These novel tools are providing an additional possibility for perioperative monitoring. However, in order to ensure that the introduction of wearable device in surgical wards does not lead to additional challenges for healthcare professionals, a careful implementation plan should be drawn up by a multidisciplinary team. In addition, a chain of liability should also be established a priori to facilitate their use and avoid ambiguity in the occurrence of a critical event.

To the editor,

New technologies are transforming perioperative medicine and hold great promise for improving patient safety [1]. In this context, innovative wearable monitoring systems are emerging. These tools are designed to continuously monitor selected vital parameters, without forcing the patient to remain in bed. In fact, they are built to be wearable, with optimized signal transmission to follow the patient’s movements [2]. Their user-friendly design and flexibility allow them to be added to the arsenal of perioperative monitoring tools, making them a viable alternative to the intermittent monitoring carried out by the nursing staff on surgical wards [3].

However, new technologies encounter significant limitations in everyday use and remain tools with great potential, but limited usability. Implementing new technologies in a system as complex as the perioperative pathway is not a simple process. Medical-legal concerns related to the not always clear responsibilities associated with the use of new technologies in healthcare are also described [4]. In our view, to promote the real use of these devices in clinical practice, it is imperative to prepare an implementation plan in advance that takes into account the different stakeholders and compliance with existing regulations, analyzing potential issues beforehand [5, 6]. This team should be made up of all the healthcare professionals usually involved in the management of the surgical patients (surgeons, anesthetists, intensivists, and nurses), together with engineers, technical staff, and members of the medical-legal staff. Once the right implementation path has been established, it is important to monitor the project’s progress on an ongoing basis. In addition, when using artificial intelligence technologies in conjunction with wearable devices, for example, to create early predictive models, software engineers should also be included in this multidisciplinary team.

Regarding the specific deployment of wearable devices for postoperative monitoring, a chain of liability should also be established a priori to facilitate their use and avoid ambiguity in the occurrence of a critical event. In this context, a hybrid management model could be advantageous (Fig. 1). This model involves initial human intervention by the nurse in the event of an alarm. A subsequent collection of additional parameters leads the nurse to decide whether to continue monitoring (e.g., in the event of a false alarm) or to alert the ward physician. In the latter situation, the clinician supplements the scenario with additional clinical information by deciding whether to continue monitoring, request specific investigations, add/change ongoing treatments, or alert the anesthetist/intensivist. It is clear from this approach that human decision-making remains central, using these devices as tools for an early identification of adverse events, but not as a substitute for human assessment. The emotional and psychological safety perceived by both the patients and the healthcare workers involved in this novel postoperative monitoring modality should also be taken into account, both during implementation and subsequent audits [7].

**Fig. 1:**
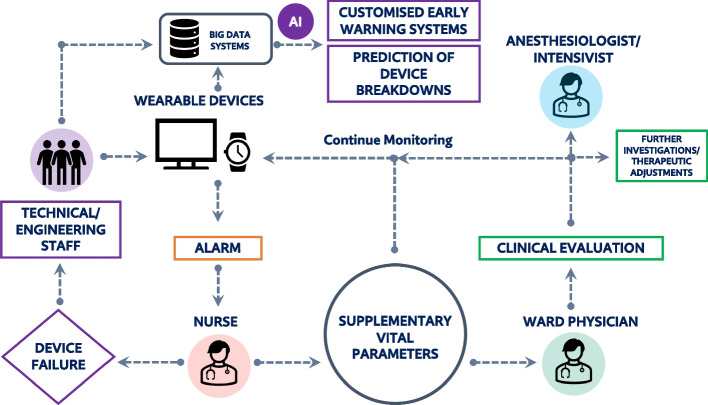
The chain of liability involved in implementing wearable devices for postoperative monitoring in surgical ward is illustrated. In the event of an alarm, nursing staff can assess whether it is a device failure or a true clinical alarm. In the latter case, complementary vital signs allow the nurse to decide whether to continue monitoring or alert the ward physician. When called, the physician will make a clinical assessment and decide how to proceed. The technical and engineering staff, on the other hand, are tasked with monitoring the proper functioning of the equipment and managing the big data system that may emerge. Moreover, through the exploitation of intelligent analysis systems, this data could also serve as fuel to finally build customized early warning systems and simultaneously to create algorithms for optimizing device performance (AI, artificial intelligence)

In conclusion, we believe that wearable devices will be increasingly used in perioperative monitoring. However, a multidisciplinary implementation project coupled with operational flow chart are key processes to make the most of the new technologies without introducing additional challenges.

## Data Availability

No datasets were generated or analysed during the current study.
